# Potential value of T1 mapping in dilated cardiomyopathy and correlation with circumferential strain

**DOI:** 10.1186/1532-429X-15-S1-P165

**Published:** 2013-01-30

**Authors:** Alexis Jacquier, Boris Maurel, Jean-Baptiste Largueze, Jean Yves Gaubert

**Affiliations:** 1 Radiology, CHU la Timone / CEMEREM, Marseille, France

## Background

The contribution of fibrogenesis to impaired cardiac function is increasingly recognized. Molli sequence is a new tool in clinical practice allowing cardiac interstitial fibrosis quantification using MRI and might bring new information comparing to delayed myocardial enhancement. The goal of this work was to assess the potential value of segmental quantification of myocardial fibrosis using T1 mapping in dilated cardiomyopathy (DCM) in comparison to late contrast enhancement imaging and circumferential strain

## Methods

This study was performed after approval of the institution's review board. Informed consent was obtained from all patients who were enrolled in this study. Seventeen patients with DCM, and 11 controls subjects were prospectively included. All patients underwent cardiac MR (Symphony TIM, Siemens, Germany) with the following sequences (parameters set according to recommendation of the SCMR): 1) cine sequence, 2) tagging sequence CSPAMM TR/TE=37.1ms/1.4ms; matrix 256, FOV 340, temporal resolution 37.1 ms, distance between tag: 6mm in mid LV slice. 3) Molli sequence (TR/TE=3.0ms/1.5ms; matrix 144x150, thickness:7 mm) acquired before and 5, 7, 9 min after gadolinium injection (DOTAREM, 0,2mmol/kg). 4) Late gadolinium enhanced sequence 10min after injection. T1 values were assessed in blood and in the 6 segments of the mid LV slice. Were calculated: R1, ΔR1, ΔR1 ratio (partition coefficient of Gd, λ). Statistical analyses were performed for patients and for segments with (+) and without (-) enhancement on LGE. Mixed linear model was used to estimate the effect of the imaging parameters of interest, allowing us to take into account the dependency between myocardial segments.

## Results

**Figure 1:**
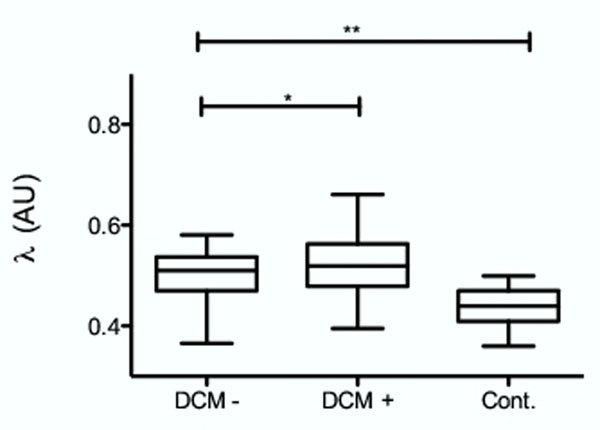
Mean lambda value for patients without any enhancement on delayed contrast enhanced images (DCM-), with at least one segment showing delayed contrast enhancement (DCM+) and control subject (Cont.).

**Figure 2:**
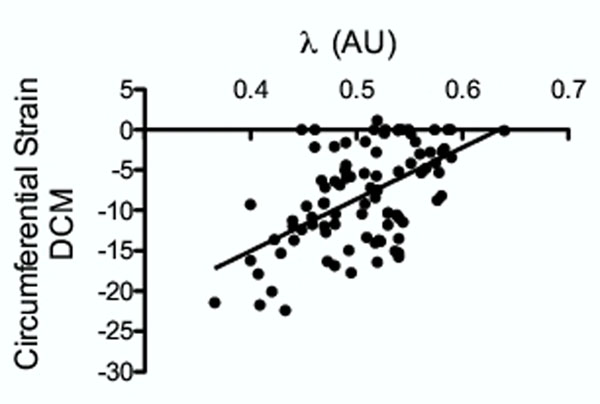
Correlation between circumferential strain and lambda in segmental analysis in DCM.

In all DCM, λ was significantly higher compared with the value obtained in control subject (p<0.0001). In DCM, λ was higher in segments showing enhancement on LGE (0.56±0.05) compared with segments showing no enhancement (0.50±0.05; p<0.0001). On the other hand λ was not significantly different between patients without (0.50±0,05) and with at least one segment showing enhancement on LGE (0.52±0,06; p=0.06) (figure 1). We found a significant correlation λ and circumferential strain in each segment (r=0.55; CI (0.39;0.68); P<0.0001) (figure 2), and between λ and LVEF (r= -0.56; CI (-0.82;-0.11); P<0.01). Using a mixed linear model, we found a significant link between λ value in each segment and circumferential strain (0.002±0.001p=0.009), as well as with ejection fraction (-0.001±0.0008p=0.04).

## Conclusions

In DCM fibrosis was a diffuse process involving all the myocardium. There was a correlation between the quantity of fibrosis and circumferential strain.

## Funding

This study was supported by a national research grant: PHRC_CMD_IRM # 2011-A00887-34

